# Sexual dimorphism of the humerus bones in a French sample: comparison of several statistical models including machine learning models

**DOI:** 10.1007/s00414-025-03417-1

**Published:** 2025-01-25

**Authors:** Manon Blanc, Siam Knecht, Kathy Nguyen, Clément Poulain, Gérald Quatrehomme, Véronique Alunni, Luísa Nogueira

**Affiliations:** 1 Boulevard Tanguy Prigent, Centre Hospitalier Universitaire de Brest, Brest, 29200 France; 2 Faculté de Médecine, Institut Universitaire d’Anthropologie Médico-Légale, Université Côte d’Azur, 28 Avenue de Valombrose, Nice Cedex 2, 06107 France; 3 Aix Marseille Université, CNRS, EFS, ADES, Marseille, 13007 France; 4 CEPAM (UMR CNRS 7264), 24 Avenue des Diables Bleus, Nice, 06300 France

**Keywords:** Forensic anthropology, Sexual dimorphism, Sex estimation, Humerus bone, Statistical models, Machine learning

## Abstract

Sex estimation is an important part of skeletal analysis and forensic identification. Traditionally pelvic traits are utilized for accurate sex estimation. However, the long bones, especially humerus, have been proved to be as effective for determine the sex of the individual.

The aim of this study was to compare the predictive accuracy of seven statistical modelling techniques including classical statistical methods and machine learning algorithms, to assess the sexual dimorphism of humerus on a French sample based on a metric analysis of 26 measurements. A total of 98 humeral bones (divided in two samples) were measured. Seven statistical models were compared: Linear Discriminant Analysis (LDA), Regularized Discriminant Analysis (RDA), Penalized Logistic Regression (PLR), Flexible Discriminant Analysis (FDA), Support Vector Machine (SVM), and Artificial Neural Network (ANN) and Random Forest (RF).

With cross validation, classification accuracy was greater than 90% (ranges between 92% and 98%) for all models without variable selection methods. The simplification of the models has improved the accuracy between 98% and 100% and also a reduction of the number of variables to 6 or less. Penalized logistic regression (PLR), Random Forest (RF) and Linear discriminant analysis (LDA) were the best accuracy models.

The measurements made at the proximal part of the humerus (WTT, CSD), at distal part (BEW, WT, MAW, THT) and of the entire bone (PLCT) stand out among the various models.

The present study suggests that the humerus is an interesting alternative for sex estimation and that non-classical statistical models can provide a new approach.

## Highlights

Analysis of humeral measurements from a contemporary French sample.

Comparison of predictive accuracy of seven statistical models.

Classification accuracy was greater than 90% for all models without selection methods.

Methods of variables selection was improved the accuracy of the models.

Relevance of new variables.

Penalized logistic regression (PLR), random forest (RF) and linear discriminant analysis (LDA) were the best accuracy models.

## Introduction

Forensic anthropology is defined as a field of physical or biological anthropology that applies the anthropological methods for the examination and analysis of unknown human skeletal remains to establish the biological profile (sex, age, biogeographical origin, stature) and determine the causes and circumstances of death in a forensic context [1–4].

Sex estimation is the one of most important procedure for developing the biological profiles because estimation of age and stature are based on sex. Sex estimation can increase the possibility of human identification by 50% [e.g., 4–4]. The pelvis is considered the most accurate bone for sex estimation with correct classifications of at least 95% [e.g., 8–10]. The sexual dimorphism of the pelvis results from different reproductive roles between males and females [1]. However, post mortem damage and taphonomic changes may prevent the collection and subsequent analysis of this anatomical region. Traditionally, the skull was considered the second-best indicator for sex assessment with an accuracy of about 75 to 90% in forensic cases [e.g., 11]. It has now been established that the use of long bones, especially the upper limb bones, provides better results [e.g. 4, 12–15].

Forensic literature has reported the use of humerus in sex estimation [e.g., 16–26] with classification percentages frequently in excess of 80%, or even 90% in more recent studies. A variation in the degree of sexual dimorphism is reported among different population [15, 19] related to the interaction of many factors such as body size, genetics, hormonal status and by the time span or biogeographical origin [19, 21].

In fact, the humerus is the largest bone in the upper limb. Because of its structure and size, it is considered one of the strongest long bones in the skeleton and is one of the long bones that have been shown to remain in better condition after death. Moreover, even in a fragmented state, it is possible to recover information from it [13, 27, 28].

Different anthropological methods have been employed about humerus dimorphism such qualitative (non-metric) morphological [e.g. 29], quantitative (metric (single, combined, index measurements) [4, 7, 19–25, 31], or semi-quantitative (scoring). More recently morpho-geometric (a modelling system that allows the combination of both methods i.e. combining shape and size in 2D or even 3D) have been proposed when traditional methods are not feasible due to excessive fragmentation of the humerus [32, 33]. Most of the humerus ‘s studies using quantitative metric [4, 7, 19–25, 30, 31] or radiographic [26, 34] methods have shown good results with correct sex assessment accuracies ranging from 68–100%. However, there are differences concerning the most discriminating measures. For some authors, measurements taken on the proximal end of the humerus are more relevant [20, 22, 25, 26]. For others, it would be the total length [17, 23, 31]. Finally, some authors have established a greater dimorphism based on measurements of the distal end [24, 32]. The combination of several measurements would show higher sensitivity results [4, 22, 23, 25, 26, 34]. Indeed, several studies show that multiple measurements and multivariate techniques offer greater validity to the biological profile assessment.

To our knowledge, the forensic literature provides rare studies using classical statistical models (except for linear or flexible discriminant analysis frequently used) based on humerus measurements [4, 12, 21–23, 26, 34]. As far as artificial intelligence models are concerned, more and more studies have been using them in various forensic fields, particularly since 2015 [35]. In the forensic anthropology field of research, AI applications have been proposed by several authors for sex estimation [14, 35–37]. Only one study [37] was found concerning the use of nonlinear methods of discriminant analysis or machine learning classifiers on humerus. However, good model accuracy was reported when humeral measurements were combined with other long bone measurements (humerus, radius, femur, tibia). When humeral measurements were used in isolation, the percentage of correct classification did not exceed 90%. Moreover, only five measurements were made on the humeral bone (maximum length, epicondylar breadth, head diameter, diaphyseal mediolateral diameter, diaphyseal anteroposterior diameter at mid-shaft) and five statistical models were tested. Finally, sex was not always known with certainty, since it could be estimated.

Therefore, the objective of the present preliminary study was to compare the predictive accuracy of seven statistical modeling techniques, including machine learning algorithms, for assessing humeral sexual dimorphism only, using a larger number of variables, in a French sample.

## Materials and methods

### Bone samples

Bones used in this experiment were acquired from French individuals (European origin) that have donated their bodies to the science between 2004 and 2019. A total of 98 left humerus bones were analyzed. A first sample, called training sample composed by 60 humerus (30 males and 30 females) (S1) were utilized to fit the models of sex estimation. A second sample, test sample, comprising 38 humerus (18 males and 20 females) (S2) were utilized in order to verify the performance of the sex estimation models. Bones were randomly distributed between the reference and test samples. In the case of body donations to science, the sex was known with certainty. A hot water maceration was employed to remove the soft tissues and obtain clean specimens. Bones with macroscopic pathological or degenerative disorders were excluded.

### Humerus measurements

**Fig. 1:**
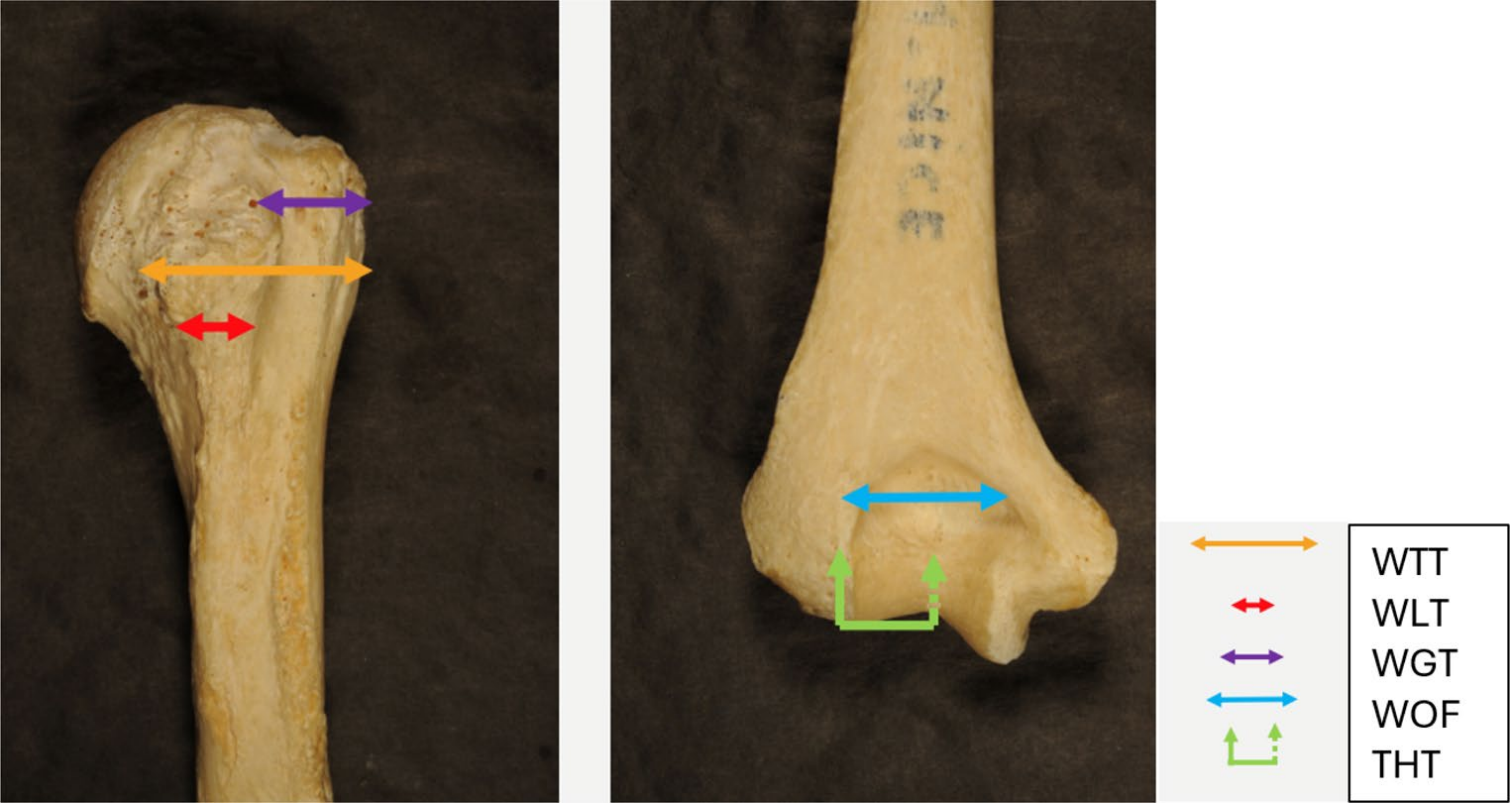
New variables in our study

The measurements were made on left humerus because it is assumed that handedness have no impact on bone size. Therefore, in any one individual, the measurements of a variable may theoretically be substituted by those of the other side depending on availability. In the literature, some studies found no significant difference in measurement according to the side [22, 24]. A total of 26 measurements were achieved. Measurements definitions were summarized in Table 1. Twenty-one of these measurements have already been described in the forensic literature. Five out of 26 were created for this study: Width between the Two Tubercles (WTT), Width of the Greater Tubercle (WGT), Width of the Lesser Tubercle (WLT), Width of Olecranon Fossa (WOF) and Thickness of Hollow of the Trochlea (THT) (Fig. 1). Five indices were also calculated from the measurements taken (Table 1).

**Table 1:** Measurements selected for this study

Variable	Brief definition	Measurement
Maximum length (ML)	Distance between the most proximal point of the humeral head and the most distal point of the trochlea, in posterior view	Osteometric board
Physiological length of medial epicondyle (PLME)	Distance between the most proximal point of the humeral head and the most distal point of the medial epicondyle, in anterior view	Osteometric board
Physiological length of lateral epicondyle (PLLE)	Distance between the most proximal point of the humeral head and the most distal point of the lateral epicondyle, in anterior view	Osteometric board
Physiological length at the hollow of the trochlea (PLHT)	Distance between the proximal articular surface of the humeral head and distal articular surface of the hollow of the trochlea, in anterior view	Small Spreading Caliper
Physiological length - hollow between capitulum and trochlea (PLCT)	Distance between the articular proximal surface of the humeral head and the distal articular surface of the hollow located between the capitulum and the trochlea, in anterior view	Small Spreading Caliper
Transversal diameter of the head (TDH)	Distance between the superior and inferior point of the head parallel to the head, in anterior view	Digital caliper
Vertical diameter of the head (VDH)	Distance between the anterior and posterior point of the head perpendicular to the long axis of the humerus, in medial view	Digital caliper
Diameter of the head with greater tubercle (DHGT)	Distance between the medial point of the articular surface of the head and the lateral point of the major tubercle, perpendicular to the long axis of the humerus, in anterior view	Digital caliper
Chirurgical neck circumference (CNC)	Perimeter of the neck at the junction between the head and the body of the humerus (constriction of the humerus located inferior to the greater and lesser tubercles) parallel to the long axis of the humerus, in anterior view	Measuring tape
Anatomical neck circumference (ANC)	Perimeter taken at the level of the zone that separates the head and the two tubercles, parallel to the head, in anterior view	Measuring tape
Circumference at sub deltoid level (CSD)	Perimeter taken at level of deltoid perpendicular to the long axis of the humerus	Measuring tape
Width between the two tubercles (WTT)	Distance from the most anterior point of the lesser tubercle to the most posterior point of the greater tubercle, perpendicular to the long axis of the diaphysis, in lateral view	Digital caliper
Width of the greater tubercle (WGT)	Distance between the most anterior and the most posterior point of the greater tubercle, in lateral view	Digital caliper
Width of the lesser tubercle (WLT)	Distance between the lateral and medial edge of the lesser tubercle, perpendicular to the long axis of the diaphysis, in anterior view	Digital caliper
Maximum diameter at mid height (MDMH)	Distance between the lateral and medial edges of the diaphysis perpendicular to the long axis of the humerus, at half of maximum length, in anterior view	Digital caliper
Minimum diameter at mid height (MinDMH)	Distance between the anterior and posterior edges of the diaphysis perpendicular to the long axis of the humerus, at half of maximum length, in lateral view	Digital caliper
Circumference at mdi height (CMH)	Perimeter taken at half of maximum length perpendicular to the long axis of the humerus	Measuring tape
Maximum diameter of the diaphysis (MDD)	Maximum diameter of the diaphysis approximately at the level of the lower third of the diaphysis, perpendicular to the long axis, in posterior view	Digital caliper
Minimum diameter of the diaphysis (MinDD)	Maximum diameter of the diaphysis approximately at the level of the lower third of the diaphysis, perpendicular to the long axis of the humerus, in posterior view	Digital caliper
Bi epicondylar width (BEW)	Distance between the most medial point of the medial epicondyle and the most lateral point of the lateral epicondyle, in posterior view	Digital caliper
Articular width (AW)	Distance from the lateral border of the capitulum to the medial border of the trochlea, in anterior view	Digital caliper
Maximum articular width (MAW)	Distance between the medial border of the trochlea and lateral epicondyle, in anterior vue	Digital caliper
Width of olecranon fossa (WOF)	Distance between the medial an lateral border of the fossa, in posterior view	Digital caliper
Width of the throclea (WT)	Distance between the medial an lateral border of the throclea, in anterior view	Digital caliper
Thickness of hollow of the trochlea (THT)	Distance between the most anterior and the most posterior point of the hollow of the trochlea, in lateral view	Digital caliper
With of capitulum (WC)	Distance between the medial and lateral border of the capitulum, in anterior view	Digital caliper
Robustness index at sub deltoid level (RISD)	Ratio between the circumference at sub deltoid level and maximum length	-
Robustness index at mid height (RIMH)	Ratio between the circumference at mid height and maximum length	-
Related index 1 (RI1)	Ratio between the maximum diameter at mid height and maximum length	-
Related index 2 (RI2)	Ratio between the minimum diameter at mid height and maximum length	-
Index of the diaphysis (ID)	Ration between the minimum diameter at mid height and the maximum diameter at mid height * 100	-

### Statistical analysis

Statistical analysis was performed using the RStudio software (version 4.2.0). Student T Test and Mann-Whitney test were used to compare each variable between males and females for S1 and S2 respectively (statistical significance *p* < 0.05).

Seven statistical prediction models were used in the present study and comprise models of discriminant analyses, logistic regression, and machine learning algorithms. The discriminant analysis included: Linear Discriminant Analysis (LDA), Flexible Discriminant Analysis (FDA) and Regularized Discriminant Analysis (RDA); the logistic regression was the Penalized Logistic Regression; and the machine learning include Artificial Neural Networks (ANN), Random Forest (RF) and Support Vector Machine (SVM).

Discriminant analyses models are used to explain and predict the probability of belonging to a group or category from measured data [38]. In our study, this statistical approach allows not only to predict sex, but also to estimate an individual’s probability of being male or female [39]. During the process of discriminant analysis, a value called *centroid* is calculated for each sex which is defined as the average value of all variables involved in the analysis for a sex group in a multivariate model. The position of the centroid is crucial in the model: for example, if the value of an individual is close to the position of the centroid, the probability that the individual belongs to this sex group is high. As a result of these calculations, a formula called discriminant function is obtained. When the values of an individual are placed in this function, the sex can be estimated depending on whether the result is larger or smaller than the cut-off point [40]. LDA is the parametric model of discriminant analysis [41]. It has been the most used model for sex estimation in forensic anthropology [e.g. 12, 19, 21, 42] and is almost the only model used in the literature to date for sex estimation from humeral bone measurements. This method recognizes a linear combination of predictor variables with separates mutually independent variables in an optimized way, and then creates a discriminant function that characterizes the differences between groups and classifies the new individuals whose group membership is not specified [43, 44]. It is a parametric classifier that models the probability of occurrence of one of two classes of a dichotomous dependent variable [44, 45]. Discriminant analysis is a multivariate parametric technique and, therefore, it is subject to several assumptions: the size of the smallest group must be greater than the number of sexual traits (independent variables), the variables must follow a multivariate normal distribution, the variance/covariance matrices of the variables must be homogeneous between the groups, while multi-collinearity between the variables must be excluded [39]. However discriminant analysis tolerates some weaknesses among the requirements, as usually it does not affect the performance of classification.

However, in the literature, these criteria were not always respected and do not affect the performance of the model. RDA and FDA are non-parametric versions of discriminant analysis [43]. FDA is a classification model based on a mixture of linear regression models, which uses optimal scoring to transform the response variable so that the data are in a better form for linear separation, and multiple adaptive regression splines to generate the discriminant surface [46, 47]. They result in a more flexible classifier that may show higher overall correct classification rates than LDA [45, 48].

Logistic regression models show the relationship between a set of continuous or categorical predictors *X = (X1*,*.*,* Xp)* and a dichotomous outcome variable Y that represents the sex of individuals [41]. This model was excluding of the present study because of the difficulties in meeting the assumptions (distributions of our variables (normality not respected) and small sample size made it difficult to obtain a stable and reliable estimate). PLR is also one of the statistical models used to predict binary outcomes. The specificity of this model is to apply a penalty to the size of the L2 norm of the coefficients, decreasing the coefficients of the least contributing variables towards zero [40].

Machine Learning (ML) is based on data mining, allowing pattern recognition to provide predictive analysis. ML is a sub-category of artificial intelligence. It consists in letting algorithms discover “patterns”, that is recurring motifs, in data sets. These data can be numbers, words, images, statistics. By detecting patterns in these data, the algorithms learn and improve their performance in the execution of a specific task [49].

Different representations or models therefore learn to discriminate groups in different ways, so that different assumptions and constraints lead to different accuracy between algorithms depending on the problem [50]. ML models are not required to respect particular statistical assumptions, such as normality, since they are based on these data [50]. Indeed, they do not operate solely on group parameters such as means and covariance matrices. Instead, many algorithms must iteratively solve optimization problems in order to find the best set of neural weights (ANN) or tree structure (RF) that provides a decision boundary, often non-linear, with a maximum classification rate [50]. Several studies in the literature were particularly interesting as they demonstrated that traditional statistical classifiers can be easily outperformed by machine learning methods [50].

ANN is an artificial intelligence model corresponding to a system of interconnected neurons that mimics the functioning of the human brain [51–53]. It is one of the main tools used in ML and can be used for sex classification and prediction. Four basic elements compose this method: an input layer, a hidden layer, an output layer and synaptic connections between the above layers [40]. When ANN is used for sex classification, the leftmost layer of the network (input layer) consists of m input units that represent the traits *X1*,* X2*,…,* Xm*, while the output layer has only one node, which is related to the sex variable. In each neural network, the input values *X1*,* X2*,…,* Xm* are transformed into an output value via appropriate weights. A crush function (usually a logistic function or a hyperbolic tangent function) allows a non-linear transformation of the input. The layers are connected to each other, and the relationship between the nodes is represented by weights. The weights of the network are adjusted by a learning process. The main parameters for building an ANN are the number of hidden layers (size) and the decay parameter. The optimization of the ANN network involves the adjustment of the size and decay parameters.

RF is a non-parametric model that allows to explain a qualitative variable from one or more qualitative or quantitative explanatory variables. Random forests are a set of decision trees. RF predict a categorical dependent variable from measurements and observations on one or more predictor variables, through a series of rules, or nodes, similar to the branches of a tree [54].

SVM are non-probabilistic classifiers that are binary in their standard form [41]. SVM aim to find a linear hyperplane separation that maximizes the distance to the nearest of each class called margins. This model in not affected by the non-equality of group variances and for some authors, SVM is said to be the most robust and accurate method among all well-known algorithms [55].

The set of statistical prediction models was constructed in two steps. First, the reference sample (S1) was used to build the different models. All quantitative variables were included in the models in order to know the overall performance of each model in predicting the probability of belonging to the male or female sex class for each humerus. Some statistical models had methods for selecting the most relevant variables. The aim was twofold: to optimize the model by increasing its performance and to simplify it by reducing the number of variables to be entered into the model.

A selection variables method was also performed to improve the models and reduced the number of the variables. In LDA model a stepwise selection and a best subset selection were used. The stepwise selection is a simple and exhaustive selection method, which predicts the best variables using a bi-directional automated procedure that use forward selections, backward selections or both combined [56]. The best subset selection is exhaustive research for the best variables that identifies the best model containing a given number of predictors [56]. This method was also used in RDA model. For the other models (PLR, ANN, RF and SVM), selection variables are not required. However, for FDA and machine learnings it is possible to show the importance of the variables, in descending order, indicating the order of relevance of each variable to achieve the accuracy.

A cross-validation method (cross-validation, k = 10, repetition = 5) was used to prevent overfitting and artificial accuracy and allowed to obtain a minimum and maximum percentage of accuracies. A second sample (S2) was also used to assess the performance of the models. In the models with variable selection methods, only the variables selected in S1 were used. For the other methods all variables were used.

## Results

Table 2 shows the statistical descriptive analysis of humerus measurements and the comparison between males and females. The measurements (average) obtained for males are higher than those obtained for females group. Statistical differences were observed in almost all variables with exception RI1 (*p* = 0.147, Mann Whitney test for independent series).

**Table 2:** Statistical descriptive analysis of the entire sample (reference and test) and comparison between males and females (Mann Whitney test, independents series)

Variables	Mean males (sd)	Mean females (sd)	p value (Wilcoxon test)
ML	333.21 (11.34)	301.58 (13.46)	< 0.001
PLME	323.38 (11.28)	292.81 (13.06)	< 0.001
PLLE	322.52 (11.18)	291.87 (12.70)	< 0.001
PLHT	327.91 (11.16)	296.96 (13.51)	< 0.001
PLCT	328.24 (11.41)	297.48 (12.76)	< 0.001
TDH	46.88 (2.23)	41.15 (2.25)	< 0.001
VDH	50.95 (2.23)	44.09 (2.23)	< 0.001
DHGT	53.95 (2.47)	46.19 (2.97)	< 0.001
CNC	113.98 (5.54)	96.00 (6.45)	< 0.001
ANC	164.08 (7.09)	143.92 (5.91)	< 0.001
CSD	71.59 (4.51)	62.15 (4.29)	< 0.001
WTT	49.69 (2.39)	43.06 (2.56)	< 0.001
WGT	37.56 (2.64)	32.58 (2.78)	< 0.001
WLT	20.15 (1.95)	17.11 (1.82)	< 0.001
MDMH	23.60 (1.90)	20.48 (1.76)	< 0.001
MinDMG	19.35 (1.37)	16.13 (1.45)	< 0.001
CMH	72.46 (4.31)	61.90 (4.59)	< 0.001
MDD	24.20 (1.48)	21.27 (1.66)	< 0.001
MinDD	20.26 (1.50)	16.87 (1.52)	< 0.001
BEW	65.14 (2.78)	55.55 (2.94)	< 0.001
AW	47.55 (1.57)	40.53 (2.51)	< 0.001
MAW	54.02 (2.29)	45.94 (2.44)	< 0.001
WOF	25.51 (1.85)	23.10 (1.67)	< 0.001
WT	26.62 (1.37)	22.48 (1.38)	< 0.001
THT	18.42 (1.41)	15.96 (1.20)	< 0.001
WC	18.48 (0.87)	15.80 (1.07)	< 0.001
RISD	21.50 (1.38)	20.47 (1.36)	0.014
RIMH	21.96 (1.54)	20.91 (1.45)	0.026
RI1	7.09 (0.60)	6.80 (0.60)	0.147
RI2	5.81 (0.42)	5.35 (0.45)	< 0.001
ID	82.11 (3.76)	78.95 (5.48)	0.024

Accuracy of different statistical models was presented in Table 3. The percentage of correctly classifications was greater than 90% using all variables. RDA was the best model among the discriminant analysis before variable selection. PLR displayed an accuracy average of 0.98. Accuracy of the different machine learnings ranges between 0.96 and 0.98. Variable selection methods were utilized to reduce the number of variables used in a final model to obtain a better performance of the different statistical models. In LDA model, accuracy increases from 0.92 to 0.98 when using stepwise method and to 0.99 with best subset selection. The number of variables used decreases to one and five (stepwise and best subset selection respectively). RDA and PLR models also increase the accuracy after selection methods. In the models without selection of variables (FDA, RF, ANN and SVM) we note that the percentage of correctly classifications is extremely high.

**Table 3:** Accuracy (average) of the different statistical models under cross-validation

Statistical model	Without selection methods (all variables included) (%)	With variables selected (%)	Method of variables selection or optimization	Variables selected
LDA	0.92	0.98	Stepwise = (direction = “both”)	WTT
		0.99	Best subset selection	PLCT, CSD, BEW, WT, RISD
RDA	0.97	0.99	Best subset selection	PLCT, CSD, BEW, WT, RISD
FDA	0.96		-	-
PLR	0.98	1	Penalization of worst variables	PLLE, BEW, MAW, WT, WTT, THT
RF	0.98		-	-
ANN	0.96		-	-
SVM	0.97		-	-

**Fig. 2:**
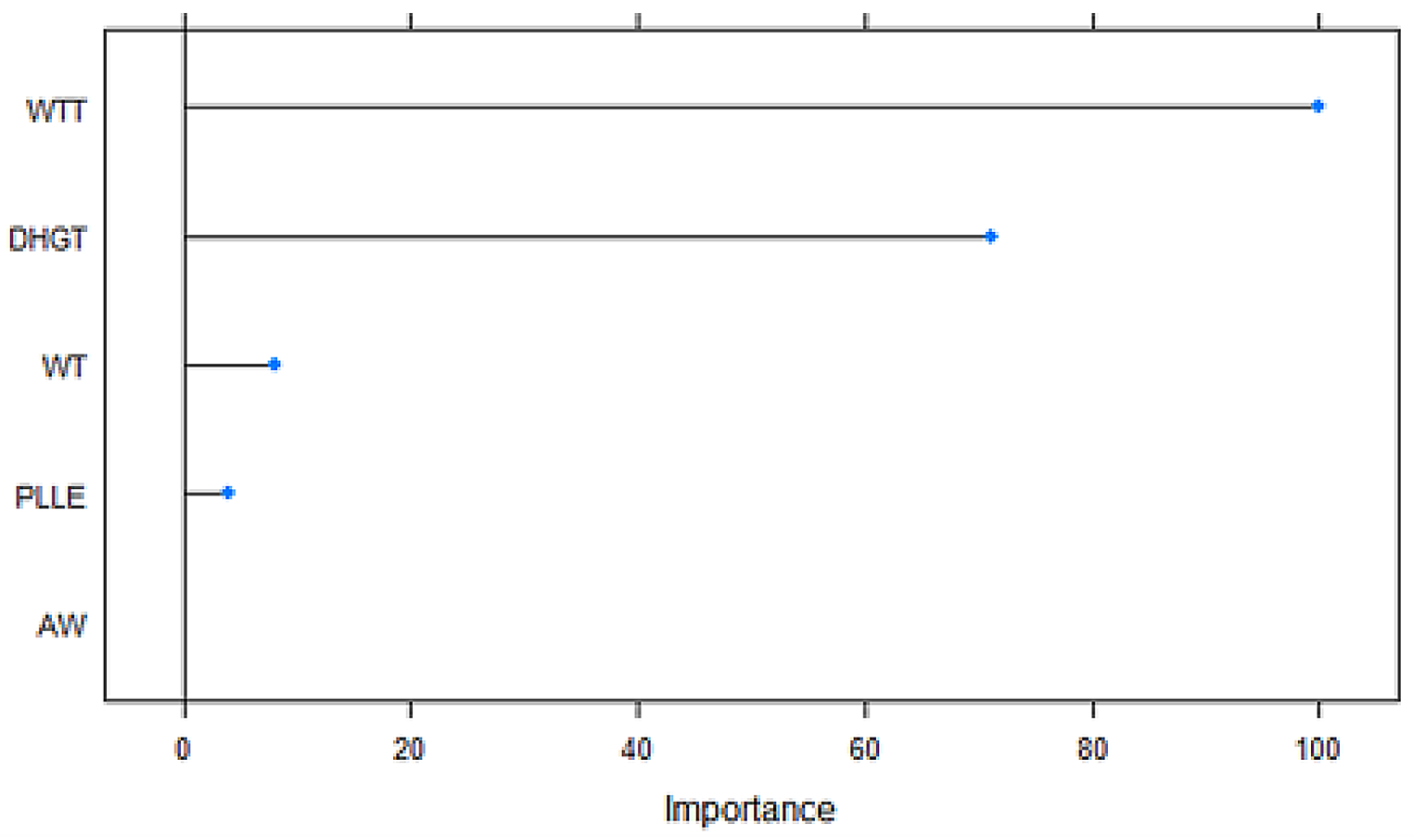
FDA’s plot

**Fig. 3:**
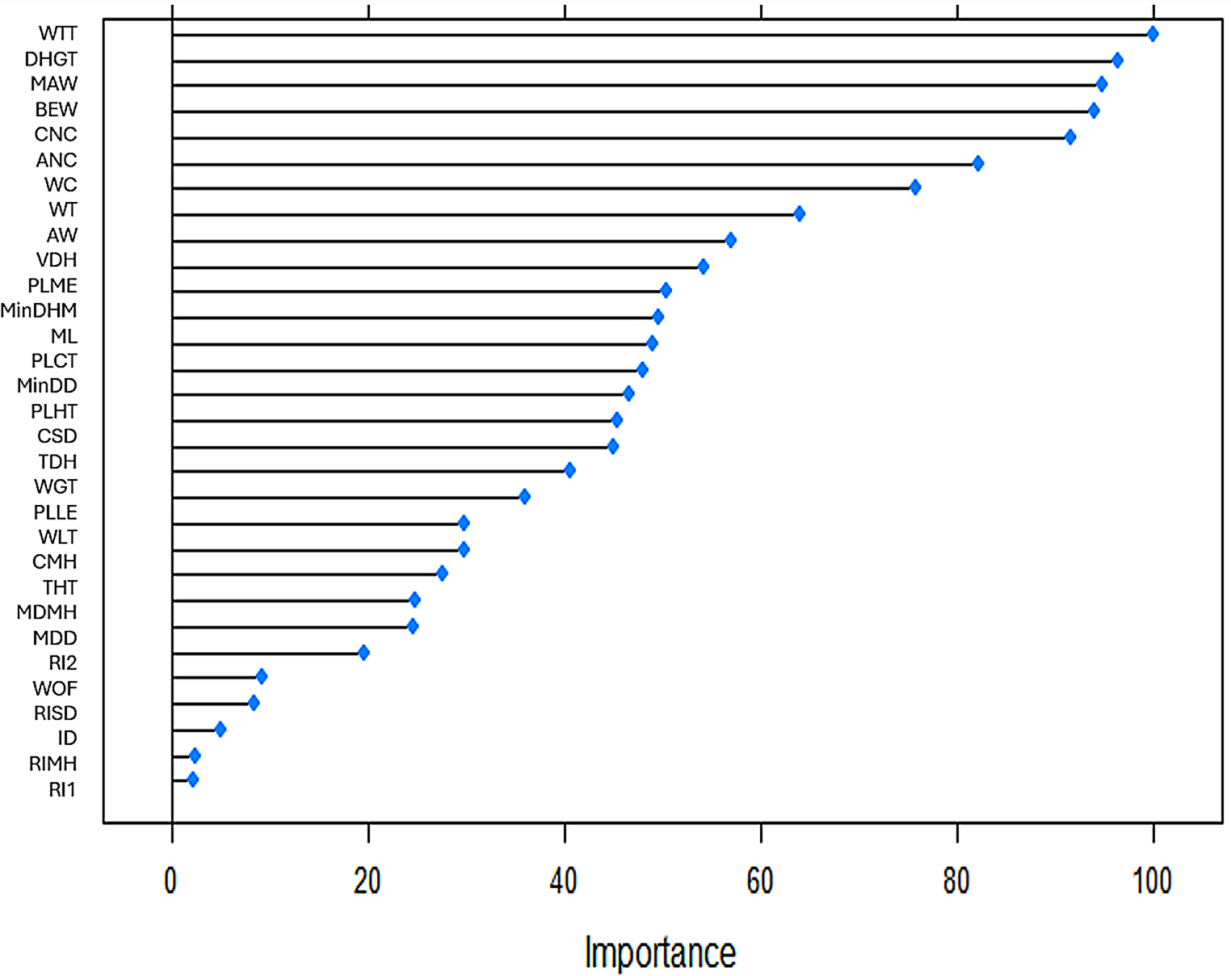
RF’s plot

**Fig. 4:**
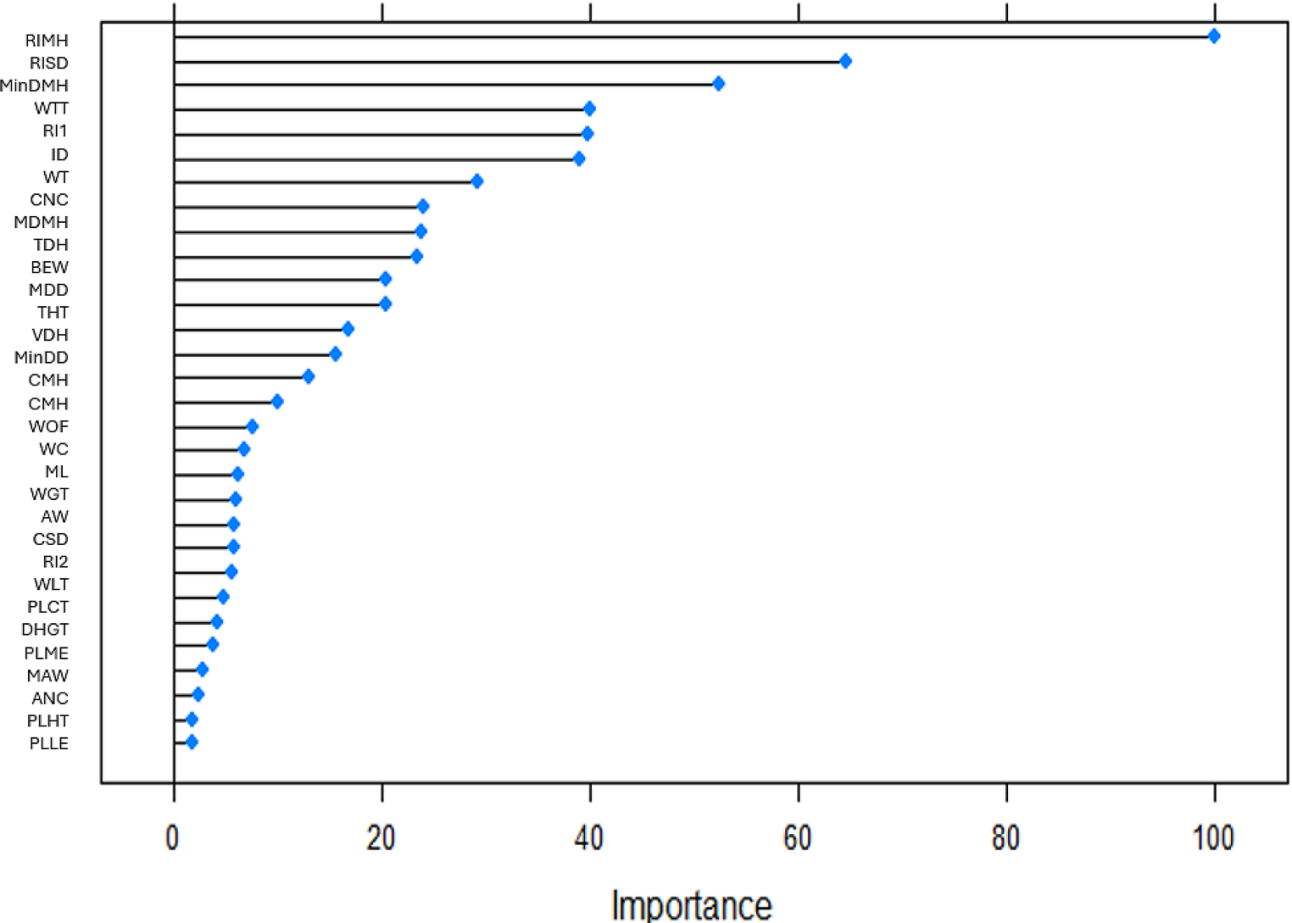
ANN’s plot

**Fig. 5:**
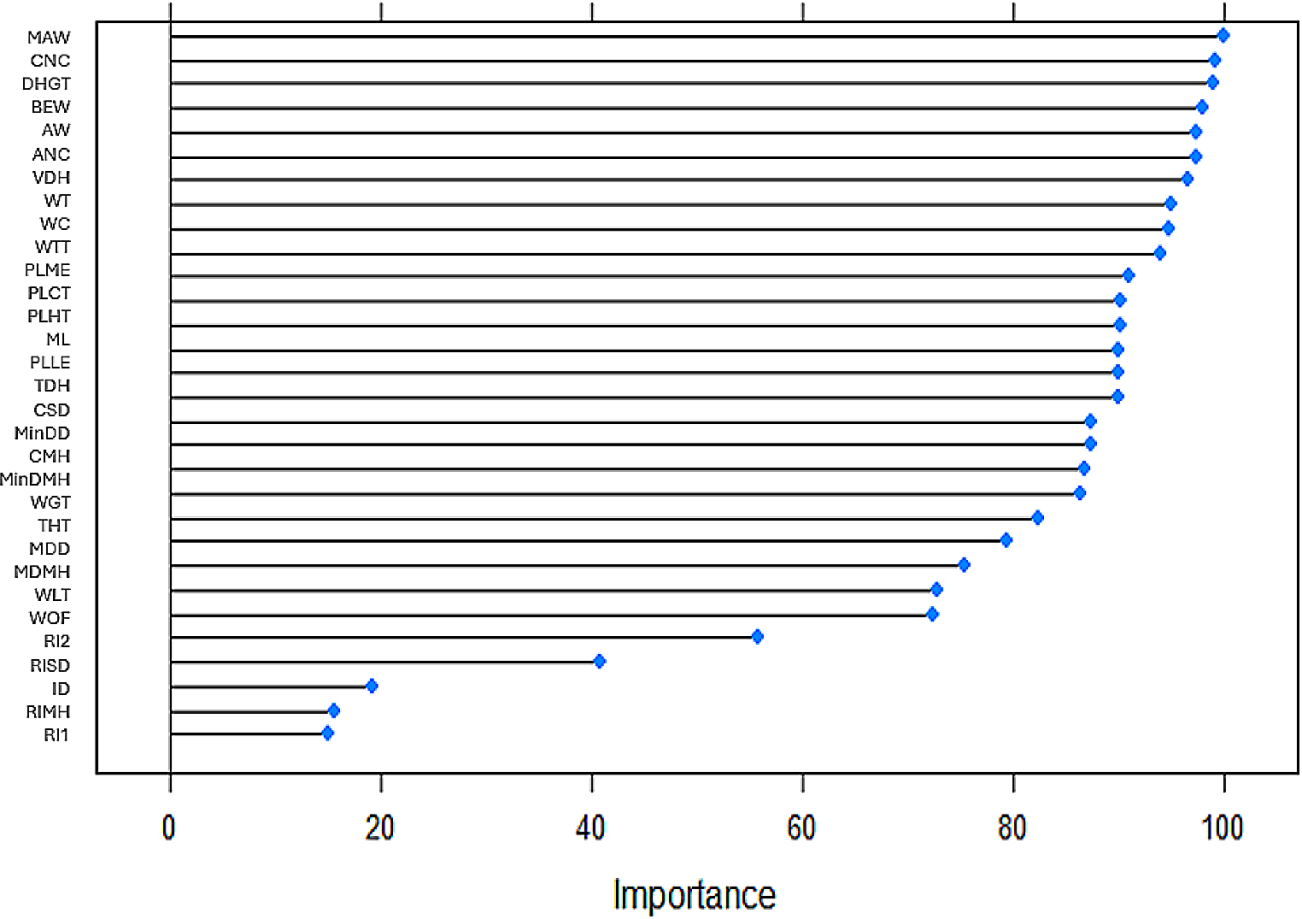
SVM’s plot

Variables WTT (new variable), BEW and WT were presented in all almost selection methods (Table 3). Figures 2, 3, 4 and 5 to R show the importance of the variable’s contribution, in the models without variable selection methods (FDA, RF, ANN and SVM). In FDA model, only four variables are important to establish the accuracy of the model, WTT, DHGT, WT and PLLE (Fig. 2). If we make the prediction on the test sample with these four variables the accuracy goes up to one. In RF model (Fig. 3), there are five variables that are important more than 90% for the success of the model: WTT, DHGT, MAW, BEW and CNC. In ANN model, almost all variables are important to the performance of the model with exception of PLLE. RIMH is the only one that stands out (100% of importance) (Fig. 4). Concerning SVM model, there are 15 variables that are important than 90% (Fig. 5). Variables WTT, BEW, WT were presented in all seven statistical models. Variable DHGT is one of the best variables in the models without selection methods.

Table 4 present the accuracy average of the different models on test sample (S2) with variables selected and using all variables for FDA, RF, ANN and SVM models. While some models predict 100% of the validation sample (LDA setpwise, PLR and RF) some models like LDA (best subset), RDA, and ANN drop off drastically and have poor predictions, close to 50%.

**Table 4:** Accuracy of the different statistical models on test sample

Statistical model	Accuracy test sample
LDA	1 (stepwise) 0.53 (best subset selection)
RDA	0.53
FDA	0.47
PLR	1
RF	1
ANN	0.65
SVM	0.84

## Discussion

Sex estimation is an important part of analysis of skeletons and forensic identification process [8]. Traditionally pelvic traits are utilized for accurate sex estimation. However, the long bones, especially humerus bones, have been proved to be as effective for determine the sex of the individual [4, 12, 19]. The morphology of the humerus is considered to be a strong indicator of sex, particularly because women are thought to have narrower shoulders than men [17, 18]. It is also explained by the fact that this area is subject to greater biomechanical functioning and stresses and by the fact that the movements of the shoulder joint are greater than those of the elbow.

In the present study, 98 humeral bones (divided in two samples) were measured. As the scientific literature provides few studies concerning the use of non-linear discriminant analysis methods or machine learning classifiers in estimating sex from humeral bone [37] in comparison with classical discriminant analysis, the aim of this experiment was to compare the predictive accuracy of seven statistical modeling techniques, including machine learning algorithms, for assessing sexual dimorphisms of the humerus on a modern French sample. A total of 26 measurements were performed. Twenty-one variables have already been described in forensic literature. Five out of 26 were created for this study.

The descriptive statistics have showed that most of the measures were higher in men. The maximum values observed in females overlap all the minimum values observed in males as demonstrate in other studies [e.g., 19, 57]. This overlap zone is therefore unavoidable because there will always be short or slender men and tall or robust women. The comparison between males and females has showed significant differences except for the RI1 (Table 2) in agreement with the forensic literature [26, 58–61]. In fact, bone remodeling differs from males and females with more cortical bone development in male adolescence. In addition, maturation and bone growth in males is later when compared to females.

### Reference sample

In the present study, seven statistical models were tested and the accuracy of which one was compared.

LDA model has produced an interesting accuracy of 92% using all variables. This accuracy was increased to 98% and 99% (depending on the variable selection method) (Table 3) with a drastic reduction on the number of variables. The stepwise method was allowed to select only one variable in LDA model (WTT). Most studies using discriminant analysis for sex determination already showed good results with accuracies of more than 80% [e.g. 21–23, 37, 62] and LDA is generally considered as more powerful when compared to non-parametric variants of a discriminant analysis [12]. However, they were also sometimes less effective [63].

Non-parametric models (RDA, FDA, PLR) that have the advantage of avoiding assumptions that are sometimes difficult to meet were also used. For the RDA model, the accuracy was high from the outset, rising from 97 to 99% after the variable selection method. The objective for this model was primarily to reduce the number of selected variables from 32 to 5. PLR was the model of non-parametric discriminant analysis with the highest accuracy and was already distinguished from other statistical models in the literature [36].

Machine learning had already proven its effectiveness in various forensic fields [35]. Thus, several studies have already indicated the potential of RF in anthropological research [e.g., 14, 37, 54]. The RF model has been described as a more efficient method than other commonly used techniques (discriminant analysis or logistic regression) [50], and it was this model that gave the highest percentage of correct classifications in the previous study on the humerus [37]. This algorithm allows to use the data to its full potential and to make objective and informed decisions. Classification trees have the advantage that they perform very well with missing data. Unlike discriminant analyses that must eliminate individuals missing even a single predictor variable, randomization forests can either remove the individual from a particular tree (or in some cases from a particular node) or use the median of the class estimate to replace the missing value; or use a proximity measure to extrapolate the missing data. Thus, not only do RFs perform better than discriminant analyses, but they also include a larger percentage of the original reference sample. Furthermore, it is interesting to note that some authors have shown that even when sample size and/or variance inequality negatively affected classification rates in more traditional methods such as logistic regression and discriminant function, classification trees such as random forest models became more accurate in comparison [64]. Finally, the RF was the simplest model as there was no need to select variables. ANN showed interesting results (96% accuracy) on the reference sample. ANNs had been shown to correctly predict sex in the literature [35, 36]. The main limitation of the application of ANN is the risk of over-fitting the model. The accuracy of SVM was 97%. SVM had also been shown to correctly predict sex in the literature [36, 37] with in some studies, such as ours, a better percentage of correct classification than ANNs [65]. The advantage of this algorithms is that you do not have to select the variables (however, you don’t know the variables used, only the order of importance).

In total, on the reference sample, the different models gave high accuracies, above 90%. For the models without variable selection (all variables included) the performance was above 95%. The variable selection allowed a drastic reduction of the number of variables.

### Test sample

The performance of LDA with best subset selection, ANN, RDA and FDA models were strong in the training sample (S1) cross validated. However, in the test sample (S2) the models performed poorly with accuracy close to 50%. That can be explained by overfitting that occurred during cross-validation, where the models were highly tuned to the training data but failed to generalize to the test data. The predictive model gives very good predictions on Reference sample data (data it has already “seen” and adapted to), but will predict poorly on data it has not yet seen during its learning phase. This problem of overfitting, which led to average results in our study, contrasts with the more promising findings reported in some other studies [36]. Thus, in the test sample, the best performing models were LDA with stepwise, PLR and RF.

Consequently, in our study, the most efficient models were LDA (with stepwise selection), PLR and RF in both test samples (S1 and S2) (Tables 3 and 4). PLR and RF models gave the best results with an accuracy of 100% at the end of the variable selection method for PLR (98% with all the variables) and 98% for RF. On the test sample, an average performance was maintained at the end of the cross-validation, since whatever the 50 cross-validations, the accuracy obtained in the end for the two models was always 100%. These results agreed with a previous study on long bones using machine learning of where RF and PLR stands out with the highest accuracy and seems to be the best models [37]. Finally, and excepting the RF algorithm, these results support the notion that classical algorithms like LDA and PLR are still highly competitive and do not underperform when compared to machine learning based algorithms, particularly when the results need to be generalized to other samples.

### Relevant variables

Selected variables differ according to the selection method that was used. Indeed, each variable selection method shows a group of best variables that are not necessarily the same from one method to another. This is not surprising, since each statistical model uses different variables to unlock the best that each model has to offer.

There would exist a tendency for the proximal segment to be more discriminating. Fasova [22] had hypothesized that proximal measurements would be more accurate because this area is subject to greater biomechanical and stress function while elbow movements would be more restricted than those of the shoulder. For the proximal epiphysis, the vertical diameter of the head would be the best parameter and offered a percentage of correct classification between 87% and 95.5% according to the authors [26, 34, 57–60, 66, 67], followed by the transverse diameter of the head [7, 20, 59], the humeral circumference [22], and the vertical diameter added to the width of the major tubercle [34]. In our study, it is indeed a variable measured on the proximal epiphysis, WTT, which appears to be very interesting, since it was selected in two of the statistical models (LDA and PLR), and even the only one selected for the LDA model with stepwise optimization, and made it possible to obtain a perfect average of 100% accuracy on the test sample at the end of the cross-validation. This variable had never been used before in the literature. On the other hand, the vertical diameter of the head and the transverse diameter of the head, the variables most cited in the literature, did not emerge from our algorithms. This could be explained by the fact that the populations from the studies cited were of different origin from ours and we know that there are important interpopulation differences in both bone morphology and bone size that do not allow extrapolation [68].

The distal end is also often cited in the literature. The most frequently cited variable is the epicondylar width, which is said to provide percentages of correct classification varying according to the authors from 68.8 to 91.62% [7, 22, 24, 26, 34, 57, 60, 69, 70]. In one study, the width of the olecranon fossa showed a high sexual dimorphism, with an accuracy of 94.0% [32]. Our results are thus compatible with those of the literature. Indeed, out of all our variables measured on the distal epiphysis, two variables stand out as being among the most relevant: BWE (in 3 models) and WT (in 3 models), offering a percentage of well classified cases, by being associated with other variables.

The maximum length of the humerus also provides good results in the literature, offering percentages of correct classification between 72.1 and 93.3% [7, 23, 26, 31, 34, 66]. Our results are therefore correlated. Indeed, PLCT (Physiological length of the proximal articular surface of the head - hollow between capitulum and trochlea) and PLLE (physiological length of the lateral epicondyle) are two of the 27 variables retained by our algorithms. LPCT is retained in two models and LPEL in one model, offering percentages of correct classification, in association with the other retained variables, between 97 and 100%.

Thus, in our study, the most discriminating variables could concern the proximal and distal epiphysis and the length of the bone, as observed by most authors.

Two variables targeting other anatomical areas, and an index stand out: PSD (CSD), RISD. CSD, rarely cited in the literature [31], is selected here in two of the algorithm models and offers a classification percentage of 99% in combination with the other selected variables. IRSD, which is the subdeltoidal robustness index, never cited in the literature, when co-selected with other variables, allowed us to obtain an excellent classification rate of 99%.

Several advantage and limitations must be mentioned concerning the sample. First, we used quantitative methods. Unlike the qualitative methods are based on the description of observational traits and are often very subjective depending on the experience of the examiner and subject to high intra- and inter-observer error, quantitative methods are based on measurements with precisely defined variables for greater simplicity, objectivity and reproducibility. Indeed, they allow the elimination of the subjectivity inherent to morphological evaluation and thus reduce inter-observer and intra-observer errors [18]. Secondly the bones were mainly from scientific donations, forensic autopsies, or anthropological expertise and all came from European individuals from the south of France. The sex was perfectly known for each individual and the population used was contemporary (recent). Indeed, variations on the bone according to the chronology called secular changes had been noted in the literature.

The small sample size was certainly perfectly characterized, but prevented generalization of the statistical models and thus constituted the main limitation of the study. In particular, models suffering from overfitting would have required a larger learning sample for better generalization.

A limit can be found in the fact that the sample was limited and came from elderly people not representative of the general population. Indeed, some studies had shown that age-related bone changes could lead to a misclassification of sex. Ageing is thought to influence skeletal morphology in different ways [29]. During the ageing process, bone resorption occurs on the cortical surface, while at the same time this process is compensated for by periosteal apposition and bone enlargement [71, 72]. Thus, young boy would tend to be classified as young girl and older women would tend to be classified as men. Thus, it is not possible to extrapolate our results to a general population and especially to the young subject. It therefore appears necessary to test these variables on a larger and more diversified sample (mixing subjects of different ages and origins) and renew the samples because the old populations are no longer a reflection of the subjects of our time.

**In conclusion**, in the present study, we compared the predictive accuracy of different statistical models for sex estimation generated from humeral bone measurements of a French sample.

The overall performance of the models is very similar, ranging from 92 to 98% or even 100% after correct classification on an independent sample and demonstrates the relevance of the humerus in sexual dimorphism. PLR and RF were the two models that stood out from the other statistical models with an average performance of 100%. RF was the simpler of the two since it did not need to use any method of variable selection. The most discriminating measures were for the proximal and distal ends of the humerus. Three variables appear to be the focus of this study: BWE and WT, which were already described as relevant in the literature, and WTT, a new variable never used before. The present study suggests that the humeral bone constitutes a valid alternative for sex estimation of skeletal remains with comparable classification accuracies to the pelvis or femur and that the non-classical statistical models may provide a novel approach to sex estimation from the humeral bone.

The relatively small sample size (*n* = 98) is a limitation of the study, but the sex of all bones was fully documented.

## Data Availability

Not applicable.
